# It was all planned … now what? Claiming agency in later life in reforming China

**DOI:** 10.1017/S0144686X16000830

**Published:** 2016-08-12

**Authors:** JIAYIN LIANG, BAOZHEN LUO

**Affiliations:** * Department of Gerontology, University of Louisiana at Monroe, Louisiana, USA.; † Department of Sociology, Western Washington University, Bellingham, Washington, USA.

**Keywords:** agency, grounded theory, historical context, pathways in retirement, reminiscence, social change, urban China

## Abstract

This study explores the social construction of agency and wellbeing among 20 Chinese urban retirees aged between 50 and 82 years old (averaging 67), with a special focus on the impact of earlier life experiences in shaping later-life pathways. Today's retirees in urban China have experienced the communist collectivist ideology during the Mao era as well as the changes to everyday life brought about by the economic transformation from centrally planned socialism to a market-orientated economy. Thereby, life in retirement for Chinese elders becomes more than just an issue of dealing with increases in discretionary time after exit from full-time work, but also one of making sense of their earlier life experiences in the midst of dramatic social changes. A grounded theory approach with semi-structured, in-depth, face-to-face interviews was used for data collection and analysis. Three interrelated themes emerged: (a) reminiscence as a mechanism of meaning-making, (b) discovery and exercise of agency in later life in contrast to a rigidly structured earlier life, and (c) varying pathways to constructing the life-stage of retirement. The findings have refuted gerontological literature and public discourse that often portray Chinese elders as passive care recipients or helpless dependants. Further, the present study has practical implications for developing policies, designing programmes and providing services to improve the quality of life for today's older Chinese people.

## Introduction

The number of people in China aged 60 and above reached 212 million by 2014, constituting 15.5 per cent of the population (Xinhua News Agency 2015). The wellbeing of the ageing Chinese population has become the centre of attention for scholars and policy makers. However, the existing literature on the wellbeing of the older Chinese has mostly focused on how the elders are being cared by families, institutions and the state. Few studies explore the agency of Chinese elders in constructing a meaningful later life and engaging in self-care, and most importantly, how their exertion of agency and arrangement of everyday life are shaped by historical contexts and social changes that they have experienced across their lifecourse.

Today's retirees in urban China have experienced a series of dramatic historical events and political campaigns during Mao's regime, and the changes to everyday life brought about by the economic transformation from centrally planned socialism to a market-orientated economy. Under the communist collectivist ideology, most individuals had almost no space for making choices over important life events such as education and career, and other aspects of personal life such as religious belief and leisure pursuit. However, the massive economic and social transformations led by the 1978 reform has resulted in not only drastically improved standards of living and increasing personal wealth, but also rising social inequality, uncertain pension security and declining role of the state in private life.

Now in retirement, as China continues to change at a rapid pace, how do these retirees construct their everyday life and make meaning out of it? How are their current arrangements in personal lives linked to the historical contexts and social changes? This meaning-making process is deeply related to how Chinese elders exert agency in identifying resources and constraints and making choices and adaptations in retirement. However, the public discourse has created what Townsend (1981) called the ‘structured dependency’ of elders. Chinese elders are often portrayed as a homogenous group of passive care-receivers; not surprisingly, the impact of early life trajectories in shaping later life wellbeing is often neglected. More importantly, without recognising the agency of Chinese elders, a mismatch seemed to have occurred between the services delivered so far and the actual needs of the older Chinese population. For instance, a large amount of resources and effort from the public and private sectors have been devoted to the development of large-scale nursing homes (Yang 2014).

### Agency in retirement

With the most attention paid to the age effect, the existing literature on ageing in China has overlooked cohort and/or period variation and experiential diversity within the social and historical contexts, and failed to address ageing-related topics in China from a lifecourse perspective. This situation is largely due to the dominance of the quantitative approach to research on ageing in China, which has entailed a lack of biographic or narrative data available for analysis. Nevertheless, a few studies have indeed demonstrated the agency of the Chinese elders. Liang (2011) identified four historically situated social contributors to a good post-retirement life in urban China among a sample of the 1950s birth cohort: formative experiences related to historical period, focus on an only child, appreciation of relative happiness and leisure without self-indulgence. Shea (2014) found self-care through exercise and a healthy lifestyle to be a way that older Chinese women could experience freedom in later life, after a lifetime of self-sacrifice; however, rather than being individualistic, this freedom emerged in balanced relations between self, family and government. Jacka (2014) called for a rich and in-depth understanding of the social construction of older rural Chinese women's experiences and pointed to the importance of using an agency framework to develop ethically and politically effective policies and programmes to improve this ‘vulnerable’ group's wellbeing.

## Sensitising framework

### The lifecourse perspective

Lifecourse is understood as an age-graded sequence of social roles and events, and the lifecourse perspective is used to guide research on human lives within particular historical and social contexts (Elder, Johnson and Crosnoe 2004). In Western contexts, the lifecourse perspective has been widely applied to examine people's ageing experiences and meaning-making in later life. In this study, we focus on three components of this prominent perspective. First, the lifecourse perspective recognises the linkage between early life experiences and later-life experiences. Early life experiences often account for subsequent life outcomes, in terms of economic wellbeing, physical health and psychological states (Dannefer 2004; George 1996; O'Rand 1996). Second, the lifecourse perspective emphasises that historical changes at the macro-level deeply impact individuals’ perceptions and behaviours at the micro-level. As Alwin and McCammon (2004: 24) insightfully put, ‘How people think about the social world around them may depend as much on what was happening in the world at the time they were growing up as it does on what is happening in the present’. Third, the lifecourse perspective treats individuals as agents who are capable of making choices and adaptations, and constructing their own life paths in response to systematic opportunities and constraints. Despite the fact that individual choices are always framed within social structures across the lifecourse (Dannefer and Kelley-Moore 2009), human beings are active and creative agents in the construction of their life worlds (Gecas 2004). The authors argue that the personal ageing experience of todays’ retirees in China must be understood in conjunction with their early life experiences, in juxtaposition with social structures and historical changes at the macro-level, and with a focus on their agency in making choices and constructing meanings.

### Historical contexts and personal lives in the Mao era

Since 1949, when the Chinese Communist Party (CCP) took over power, the state had created an institutional and moral environment which greatly limited individual choices over important life events including education and career. Building a ‘classless society’ (Chen, Yang and Liu 2010) or an ‘egalitarian society’ (Whyte 2012) was the social ideal. The collectivisation of the economy and the elimination of most private property left no room for economic motivation for the individuals. Jobs were assigned by government administrators; occupational mobility was a rare opportunity. Workers believed they were holding the unbreakable ‘iron rice bowl’, a metaphor for job stability. Income was calculated according to working years rather than professional achievement. Although differences in quality of life existed between rural and urban areas, between cadres and workers, and among people of varying class backgrounds, inequality was not as significant as we see in today's Chinese society. As the entire nation struggled in deep poverty, social inequality was low, reflected not only in income and wealth but also in health and social services (Chen, Yang and Liu 2010; Wang 1995).

Further, consolidation of political power, not economic development, was the focus of the CCP during that historical period (Chen 2002). The currently ageing population had been involved in a series of radical political movements (*e.g.* the Cultural Revolution). The ‘privileged’ young intellectuals were sent down to the countryside for manual work to be ‘re-educated’ by peasants during the Cultural Revolution. As noted, schooling was interrupted for the children of the Cultural Revolution; about one-third of the urban youth who entered the labour force between 1967 and 1978, most graduating from junior and senior high school, were forced to work and live in rural areas under the send-down policy (Zhou and Hou 1999).

In other areas of personal life, including religion and leisure, individual choices had been dwarfed given the control of the state. During the Mao era (1949–1976), embracing the Marxist teaching that ‘religion is the opium of the mass’, the CCP strictly enforced atheism through several campaigns against all forms of religion (*e.g.* Christianity, Daoism and Buddhism). In addition, leisure was highly politicised. The state regulated the length, the forms and the content of all leisure activities for the Chinese people (Wang 1995). Leisure was considered merely ‘passive relaxation and restoration of energy for work’ (Wang 1995: 153). Given the collectivist doctrine, leisure activities often took place in the form of group action, and hobbies for individual pleasure were considered ‘bourgeois’ (Wang 1995).

### Historical contexts and personal lives in reforming China

Today's Chinese elders have experienced many tumultuous changes across their lifecourse. First, when economic reform gradually swept across the entire nation from 1978, their lives took more twists and turns with the state's shift from centrally planned socialism to a market-orientated economy. The economic security of urban workers formerly guaranteed by job tenure and the work unit-based welfare system has been sacrificed for efficiency in the national economy (Chen 2002). The secure employment, housing, health care and retirement benefits enjoyed by China's urban population in the pre-reform era have mostly disappeared (Chen, Yang and Liu 2010). The traditional ‘iron rice bowl’ became the ‘clay rice bowl’ – easily destructible. The number of laid-off workers from the previously state-owned enterprises reached a peak of 6.572 million in 2000 (China's Ministry of Human Resources and Social Security 2005). People who had spent most of their working years under the collectivist system had to adapt to the rapid shift to the market economy, especially during the privatisation of urban enterprises that took off in the 1990s. Nowadays, due to the privatisation of housing and medical systems, the skyrocketing housing prices and cost of living in urban China, as well as heavy medical costs, have become critical issues in people's daily lives.

Second, almost synchronising with the open-door policy, the state took a coercive move on Chinese families – with the one-child policy first outlined in 1979 – as Chinese leaders decided that controlling the population size was the best way to foster the modernisation of the nation. For people who started a family after 1979, their personal lives in relation to family lives were fundamentally shaped by the one-child policy. On the one hand, the empty-nest family and unavailability of familial care in old age is now becoming a social phenomenon, partially due to such a structural restriction encountered in their earlier lives. On the other hand, long-term care facilities and services are still underdeveloped to meet the diverse needs of the current ageing population in China.

Third, as a consequence of the economic transformation, income inequality has become a rising concern in contemporary China (Chen, Yang and Liu 2010; Whyte 2012). One example is the dual pension system, which caused people of the same educational background, professional title, position and skills to be treated differently in terms of pension income. People who retired from government organisations and public institutions receive much more compensation than those who retired from enterprises. Not until recently did the Chinese government officially announce its agenda on unifying the dual tracks within its urban pension system (Information Office of China's State Council 2015).

Fourth, the state gradually retreated from intervening in private life. Individuals were granted more freedom to choose where to live and work, how to play and, increasingly, what to believe. Not only were people able to choose a variety of leisure activities based on their own interests and socio-economic status, they were also given more freedom to form voluntary associations according to their hobbies, such as music, dance, modern drama, folk arts and calligraphy (Wang 1995). Meanwhile, other social changes in China, such as the two-day weekend policy which came into force in 1995, the emergence of personal computers and the internet in the 1990s, and the increased opportunity to travel abroad, continue to affect Chinese people's everyday lifestyles. In recent years, organised religions have also re-entered people's private lives, though the CCP members continue to be prohibited from holding religious beliefs. As Shakya (2009: 17) put, ‘the party is willing to tolerate the emergence of religion as a purely private experience, but it is not willing to see religion expressed as a sort of collective authority and a collective assertion’.

Historical contexts and social changes, as well as cultural and political ideologies, can shape people's perceptions and choices, thus affecting the lifecourse. For today's retirees in urban China, their earlier lives, on the one hand, were highly politicised and moralised with limited personal choices; on the other hand, they experienced a strong sense of job security and welfare support. Later in life, they experienced the growing economic prosperity, the changing political and moral landscape brought about by privatisation and commercialisation, as well as expanding depoliticised space for leisure and other aspects of private life. It seems reasonable to argue that life in retirement for Chinese elders is more than an issue of dealing with increases in discretionary time after exit from full-time work, but also one of making sense of their earlier life experiences in the midst of dramatic social changes. Thereby, using rich narrative data, the present study aims to continue to fill the knowledge gap. Illuminated by a lifecourse perspective, we attempt to explore the following two questions: (a) How do urban Chinese retirees construct their later life and make meaning out of it? (b) How are their post-retirement experiences linked to past and present socio-historical contexts?

## Design and methods

We used the grounded theory approach (‘a general methodology for developing theory that is grounded in data systematically gathered and analyzed’; Strauss and Corbin 1994: 273) with the aim of developing explanatory theories of basic social processes viewed in a certain context (Starks and Brown Trinidad 2007).

### Data collection

The first author received approval from the University Institutional Review Board and conducted face-to-face, in-depth, semi-structured interviews in Xi'an,^1^ Shaanxi, China during the summer of 2011. The recruitment eligibility criteria were: a retiree aged 50^2^ and above, residing in Xi'an City and with good self-perceived health. The rationale was that provided the participants had good self-perceived health, chronological age would not be the sole or predominant determinant of lifestyle chosen in retirement as ascertained early in the interviews. Probes provided in the interview guide included: Could you describe your typical day of retirement life? What makes a good day for you, and what makes a day ‘not so good’? How do you think about your current life and what would you say put you on the path that led to your thinking like this?

Purposeful sampling, or theoretical sampling, was adopted. The first author selected retirees whose conditions varied (*e.g.* occupation, living arrangement) to maximise discovery and exploration of as many dimensions of the research topic as possible and to expand the specific research focus into a broader theory. Data gathering was halted when repeated themes or issues related to the research question were heard, richness in each category was achieved and a theory line was established. The interviews lasted from 1.5 to 4.5 hours, averaging three hours per person. The first author spoke Mandarin Chinese in the interviews, while participants used either Mandarin or their own (mostly Shaanxi) dialects. Each interview was audio-recorded with their informed consent. Then the Chinese texts were transcribed verbatim for analysis and translated into English. To ensure translation accuracy, three interview transcripts chosen at random were discussed with a bilingual doctoral student in social science whose mother tongue is Chinese. To protect the privacy of the participants, pseudonyms were used throughout.

### Participants

Theoretical sampling aims to develop a theory, not to represent a population (Charmaz 2002). Typical sample sizes for grounded theory research range from ten to 60 persons (Starks and Brown Trinidad 2007). The participants in this study consisted of ten women and ten men, ranging in age from 50 to 82 (averaging 67) (*see* Table 1). Five people were born between 1929 and 1939, ten were born between 1940 and 1950 and five were born between 1951 and 1961. A few internally^3^ retired first and then fully retired five years later. All participants had a stable pension except one woman who was dependent on her husband. Eight were workers who served state-owned enterprises, with a pension income ranging from 1,400 to 1,850 yuan.^4^ The pension ranged from 2,000 to 3,000 yuan among the other occupation holders who worked in state-owned banks or public institutions such as schools. The five with the highest monthly pension (4,000–18,000 yuan) were cadres (*i.e.* CPP officials), highly skilled professionals such as engineers and doctors, or someone working for a typical monopoly trade such as the power industry. It should be noted that some retirees had chosen to return to work part-time, which reflects one of the approaches to constructing a life after formal retirement and/or the receipt of a retirement pension.

**Table 1.** Participants’ demographic profile

Name	Gender	Age	Marital status	Education	Religious affiliation	Retirement status	Occupation	Residence	Children	Grand children	Health concern	Monthly income (yuan)
Tang	F	82	Married	College	NA	Officially retired at 65 and now working part-time	Physician	With husband and grandchild (the younger son's child)	Two sons	Two	Back pain caused by a slipped disc	6,000
Su	M	74	Widowed and co-habiting	Junior High School (evening college)	NA	Retired at 60 and now teaching at a technical college	Administrative staff in a factory	With co-habiting partner	Two daughters (former wife's) and one son (current partner's)	Three	NA	4,100
Zheng	F	64	Widowed	High School	NA	Internally retired at 50, fully retired at 55, and now working part-time	Factory worker; services section (military spouse)	Living alone	One daughter and one son	None	NA	1,500
Dai	F	56	Married	Junior College	Buddhist	Internally retired at 45 and fully retired at 55	Cashier at a bank	With husband, daughter, son-in-law and granddaughter	Only daughter	One	Hypertension and blood supply problem caused by menopause	2,500
Chang	F	50	Married	High School	NA	Exited from work in 1986 and quit her second job in 2001	Staff at a pharmaceutical factory (five years) and contract worker at a state-owned insurance company (seven years)	With husband and daughter	Only daughter	None	Coronary bypass surgery	0
Luo	F	55	Married	High School	Buddhist	Internally retired at 45 and fully retired at 50	State-owned factory worker	With husband and daughter	Only daughter	None	NA	1,400
Wei	M	66	Married	College	NA	Left the position at 53, fully retired at 55, and now serving the community	Administrative staff at a professional school; pharmaceutical factory; self-employed; director of community committee	With wife	Two daughters	One	NA	2,300
Yang	M	64	Married	Vocational Secondary School	NA	Retired at 60	Bank clerk (managerial personnel)	With wife, son, daughter-in-law and grandson	Only son	One	Diabetes and reduced hearing due to childhood tympanitis	3,000
Peng	F	66	Married	Vocational Secondary School	NA	Retired at 55	Administrative staff at a state-owned shopping mall; staff at the foreign exchange merchandise supply station	With husband, son, daughter-in-law and granddaughter	Only son	One	NA	1,800
Ma	M	74	Married	Vocational Secondary School	NA	Retired at 60 and now working part-time	Senior engineer at a design institute	With wife	One son and one daughter	Two	NA	10,000
Ni	F	77	Widowed	Vocational Secondary School	NA	Retired at 45	Factory worker (transferred from cadre)	Alone	Three daughters and two sons	Five	Hypertension and heart problems	1,850
Wu	M	67	Married	High School	Buddhist	Internally retired at 53 and fully retired at 60	Logistics staff at a professional school	With wife	Two sons	One	NA	2,000
Gao	F	57	Married	Junior College	NA	Retired at 55 and now working part-time	Accountant	With husband, daughter, son-in-law and grandson	Only daughter	One	NA	2, 600
He	M	70	Married	High School	NA	Internally retired at 55, fully retired at 60, and now working part-time	Military representative in a factory; cadre at the Geology Bureau	With wife	Two daughters	Two	Coronary bypass surgery	4,000
Ai	F	80	Married	Junior College	NA	Retired at 55	Middle school teacher	With husband	Two sons	Two	Use of hearing-aid	3,000
Cao	M	64	Married	Primary School	NA	Internally retired at 55 and fully retried at 60	Factory worker	With wife, son, daughter-in-law and grandchild	Two daughters and one son	Three	NA	1,800
Li	F	63	Married	High School	NA	Fully retired at 50	Bus driver	With husband	One son and one daughter	One	NA	1,400
Kong	F	66	Widowed	Junior High School	NA	Retired at 49 (chose to retire one year earlier)	Administrative staff at a bus company	With daughter and grandson	One son and one daughter	Two	Birth hypertyreosis	1,400
Bai	M	60	Married	Junior High School	NA	Retired at 60	Managerial staff at the Provincial Electric Power Company	With wife	Only son	One	NA	18,000
Fan	M	65	Married	High School	NA	Internally retired a 55 and fully retired at 60	Machine maintenance worker	With wife	One son and one daughter	None	NA	1,500

*Notes*: M: male. F: female. NA: not applicable.

### Data analysis

The first author used memoing, a key process in grounded theory methodology (Strauss and Corbin 1998), to track her evolving thoughts during the interview and analysis. The systematic coding procedures suggested by Strauss and Corbin (1998) were followed. ATLAS.ti 6.2 software was used to assist in organising data and managing codes.

Open codes (*e.g.* reminiscence on historical experiences, the planned-economy system, lack of choice in earlier life, appreciation of social change, collectivist value, traditional education and old doctrines, making a choice or an adaptation, meaning of retirement, health awareness, family relationship, care-giving responsibility, personal leisure, constraints of social structures, selective attitude towards activities), summarising and describing each sentence of narratives at a higher level of abstraction yet staying close to the data gathered, were generated. Using a constant comparative method (Strauss and Corbin 1994) helped to compare different dimensions and properties of each category and to make it both thick and dense. The process of axial coding arranged the data in a new way that helped group the initial codes by its similarities, identify different levels of conceptualisation and discover the relationships between emerging categories. The types of categories at this stage (Strauss and Corbin 1998) included the central phenomenon (meaning construction in retirement), causal conditions (resources and constraints), context (social change, historical context), intervening conditions (demographic characteristics), strategies (reminiscing, and making choices and adaptations) and consequences (varying pathways of navigating retirement). From there, the foundation of the theoretical framework was formulated. As analysis continued to carry on across the data-set, the strategy of selective coding was applied. The core categories and relationships that helped understand and explain the phenomenon and answer the research questions were identified, and three essential themes were developed, as illustrated in the following section of findings. Variations in the exertion of agency and the way of meaning construction were compared among the informants with different demographic and socio-economic characteristics. In addition, discussion between the two authors was extremely helpful for providing alternative insights throughout the analysis.

## Findings

**Figure 1.**
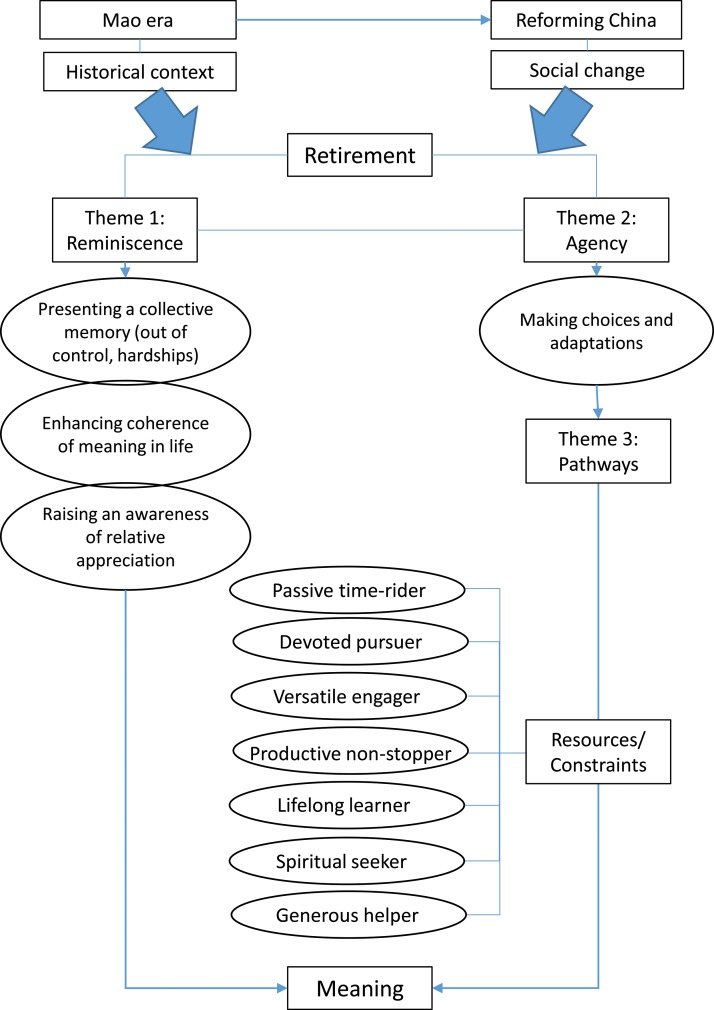
The theoretical model that explicates the social process of meaning-making through claiming and exercising human agency among Chinese retirees in contrast to their rigidly planned earlier lives.

The theoretical model is presented to summarise the findings and elicit discussions (*see* Figure 1). Three interrelated themes emerged, as elaborated below.

### Theme 1: Reminiscence as a mechanism of meaning-making

All participants, regardless of differences in cohort and other characteristics, reminisce about the past in contrast to the present. The following historical events that were significant in limiting choices of education and career in their earlier years are identified: the Korean War (1950–1953), the Great Leap Forward (1958–1961), the China–Soviet Split (1960–1989), the Cultural Revolution (1966–1976) and the ‘send-down’ policy (beginning in the 1950s and ending in the late 1970s). For example, being involved in wars earlier in life has become an essential part of Mr Ma's (74) identity in later life. He likes literature and television programmes that arouse his feelings of old times and he thinks people of his age all share this characteristic. Ms Tang (82) recalls how her earlier life trajectories of education and work were designated by the state and interrupted by the historical events:

I was originally assigned to assist the war and go to Korea, but the war stopped, so the Ministry of Health asked us to come to Beijing and stay there … In the early post-Liberation days, the state encouraged studying in the Soviet Union … We studied [Russian] for more than a year and passed the exam to study abroad, but in the end, we were not able to go, because the relationship between China and the Soviet Union was then broken in the 1960s.

Ms Tang's past is bound up with her present self. Now in her eighties, she revisits her past experiences by not only retelling her stories verbally but also planning a trip to Russia soon. Mr Mai (60) recalls his experience of ‘going to the countryside’. Every year after retirement, he travels to visit Yunnan Province where he was ‘sent down’ to visit some old friends and seek memories. Besides their own restricted earlier lives, some informants from the older cohort also reflect on how their children's life choices were limited, a difference from the younger cohorts. Ms Ni (77) recalls the days when she was working in a factory and the historical event of standard rationing with food stamps. She recollects when her children were having a hard life during the ‘Up to the Mountains, Down to the Villages’ movement.^5^ She retired at a young age, as her work position was inherited by her daughter under Deng Xiaoping's^6^ call-back of the ‘educated urban youth’.

Rather than lingering on the surface of collecting memories, through reminiscence, the informants endeavour to make sense of their past and enhance coherence of meaning in life. Ms Chang (50), the youngest, evaluates her earlier life in a similar way to the older cohorts: little room for self-choice, no conception of individual dreams and lack of freedom to define one's own life. She recognises that the education and ideology which she grew up with are influential on her later-life goals:

Sometimes I feel that *I didn't have any dreams when I was young* anyway … I think it might be the different education we received when we were young. At that time, it seemed that we had no idea about what dreams or ideals were, so we had nothing to pursue in mind. Now my financial condition is good and I do have time, but *I can't imagine what dream I want to accomplish*.

More importantly, although some informants recognise how the planned economy and collectivist ideology suppressed their aspiration for personal desire and dreams, for most of them, a difficult and choiceless past does not translate to a difficult and choiceless present. Rather, it helps them stimulate a positive evaluation of their current life. While ‘depressing and horrible’ scenes from her life during the Great Leap Forward^7^ and the Cultural Revolution arise vividly in Ms Kong's (66) mind, she shows great appreciation of social change and the improved standard of living nationwide since the 1978 reform. Her past experiences serve as a reference point for evaluating and appraising her life in the present: ‘Because I have experienced too many hardships, I feel today's life is really good and I value it very much.’ Mr Ma (74) sees retirement as bringing time for enjoyment as a reward for those previous hardships: ‘I feel that my life, just in the last few years, has been the best in my life … and *I deserve it*.’ Ms Ni (77) also comments on how blessed she feels with the present due to the difficulties she endured and the resilience she cultivated over the lifecourse: ‘Since I had borne hardships in the past, no matter what difficulty I would have now, I'm not afraid of it, and I can overcome it.’

As demonstrated above, actively engaging in reminiscence to link the past with the present is universal across the three cohorts. Through the process of reminiscing, these retirees present a collective memory (out of control, hardships), enhance coherence of meaning in life and raise an awareness of relative appreciation. As stated in the lifecourse perspective, early life experiences often account for various subsequent life outcomes (Dannefer 2004; George 1996; O'Rand 1996), and macro-level historical changes have an impact on micro-level individual experiences (Alwin and McCammon 2004). For the Chinese elders, their positive psychological states in retirement today could be traced back to the resilience and endurance they have cultivated in their earlier years during the Mao era of the centrally planned socialist system. Seeking balance and harmony in their life through the strategies of relative appreciation and historical comparison, rather than simply following a dualistic notion of happiness and unhappiness, is what characterises their mentality in old age.

### Theme 2: Discovery and exercise of agency in later life

Using reminiscence as a way of making sense of their past and treating the earlier hardships as strength to build and reinforce their character demonstrates a dynamic process of discovering and exerting agency in later life among the informants. Such agency is also manifested through the choices and adaptations they have made in the areas of activity and technology – things not allowed or unavailable in their earlier years. Mr Bai (60) insightfully explains the importance of being proactive: ‘I feel living in this society is actually about *making adaptation*s … whether you actively catch up with it or you passively follow it.’

These retirees are cognisant of the challenge of population ageing, especially with the only-child generation, that familial care is dramatically declining within the current family structure. As a result, engagement in self-care and socialising with peers through exercising outdoors has become a common activity in retirement. The park, or public square, is the most popular social venue for older Chinese, especially those from the working class, to get together and enjoy outdoor activities. Easy access to open space seems critical for smoothing retirement transitions. Living close to a park has made it possible for Ms Zheng (64) to stay engaged with others: ‘There are many people like me in our residential compound having nothing to do. Fortunately, we have a park nearby.’

It is found that hobby-based leisure activities can be important and empowering resources in helping the informants transcend the low point in retirement. Mr Wu (67) was feeling ‘directionless’, ‘anxious’, even ‘abandoned’ after retirement due to work-role loss, especially given how structured life had been throughout most of his life; however, he adapted through engagement in hobby-driven leisure pursuits and religious practice, activities that were prohibited during his earlier years. Ms Li (63) started attending senior university after retirement to make up for her unrealised college dream in youth. Although she had never painted before, she is now a skilled artist in retirement. Mr Yang (64) loves making and fixing stuff by hand in retirement. Although he portrays himself as a passive time-rider, his ability to do something useful is translated into tangible activities wherein a sense of control and achievement is exhibited. Importantly, he thinks this hobby in retirement grows out of the survival skills cultivated in his earlier life when poverty and hardships were prevalent: ‘It's not how capable I am, but most people like me who are from a poor family can make stuff by themselves.’

The prevalence of personal computers and the internet since the 1990s, and the emergence of new technology (*e.g.* smartphones) and social media (*e.g.* blogs), continue to affect the everyday lifestyles of older Chinese, similar to their counterparts in other regions of the world. They seem to respond in three ways: contemptuous, indifferent or appreciative, after a careful assessment of the pros and cons. Mr Wu (67) is not interested in watching television or reading newspapers, as he thinks information provided by mass media is fake and worthless. He chooses to stay away from worldly affairs in general in order to avoid distractions and feel peaceful at heart, using the internet only for looking up Buddhist materials online. Mr Bai (60) sees the internet as a way of acquiring information and knowledge, but is critical of the virtual world: ‘I felt people in the virtual world were pretty individualised, and sometimes I couldn't accept what they posted.’ For Ms Gao (57), social media has become something essential that gives meaning to life in today's hustle and bustle: ‘people who have jobs are all very busy, so Qzone [blogging] becomes a platform for communication’. Another internet user, Mr Ma (74), surfs online to find information, browse news and book train tickets. Though Pinyin (transliteration of Mandarin Chinese into the Latin alphabet) makes typing difficult for him, he has adapted by using a writing pad. Ms Ai (80) is aware of the abundance of technology – she takes pictures of her daily life with a digital camera and uses a cell phone with bigger fonts designed for the elders to communicate with family and friends – but she refuses to use computers due to fear of radiation and adverse health effects, a common concern shared among the informants.

While the influence of social changes is widespread at the macro-level, their impact on individual lives in retirement differs at the micro-level, depending on the individual's personal belief, educational and work experience, family background and leisure preference. Ageing experiences rest on the dynamic interplay between structure and agency (Dannefer and Kelley-Moore 2009; Gecas 2004). In the present study, bound by social and historical structures, human agency is reflected in the process of making choices and adaptations, not only in terms of activity participation and technology use, but also regarding how to construct the life-stage of retirement based on one's resources and constraints.

### Theme 3: Varying pathways to constructing the life-stage of retirement

Through reminiscence of the past and exertion of agency in the present, the retirees navigate their own pathways in later life. Seven qualitatively different pathways were identified by the researchers: passive time-rider, devoted pursuer, versatile engager, productive non-stopper, lifelong learner, spiritual seeker and generous helper. Each participant shares the characteristics of one or more pathways. Except for the passive time-riders whose dormant agency seems not to be revived yet, most of the informants demonstrate how they discover and exercise agency in mapping out their retirement years, in relation to their earlier experiences.

*Passive time-riders* are those who feel themselves being put in a passive position in dealing with the abundance of discretionary time in retirement. For some informants, earlier life experience in the previously planned system is deep-seated and has influenced their thinking and behaviours in old age: they are unaccustomed to making personal choices on their own. People falling into this pathway easily bring out a passive attitude towards life in general and do not see their daily life experience as meaningful since those activities engaged in are not proactively pursued driven by their own choices. Six informants describe themselves as passive time-riders. Among the three women, two are widowed; being widowed seems to narrow down their social network, thus reducing their opportunities of meaningful engagement. Four are retired workers living on a low pension, while the other two have a moderate or high income. The lack of career choices, financial strain and health problems in early life, compounded with entrenched traditional values and morality, may have instilled a strong sense of constraints. The passivity was transferred from earlier life to later life.

Mr Yang (64) focuses more on the limitations instead of the potentials that he may explore. He identifies things which he can engage with but no further action is done. Though reduced hearing may limit and reduce his social interaction, he also ascribes it to his passive attitude. Ms Dai (56) idles away by expanding ordinary routines to fill up her time reluctantly, and things presently engaged in are often influenced by others and not as personally self-identified as liked and wanted. She perceives financial strain as a barrier to having a satisfactory life; however, she forwent a re-employment opportunity as an accountant for she believes ‘such work is all about tax fraud’, which challenges the ‘orthodox education’ with which she grew up. Her entrenched moral values conflict with the current economic environment and diminish her ability to make a living. When confronting social change, some choose to accept differences and adapt, whereas others stick to their old niche of values and morality.

*Devoted pursuers* are those who are actively engaged in hobby-based activities with high devotedness and have developed professional skills along the way. These people perceive retirement as an opportunity for leisure pursuits. Six informants belong to this category, three men and three women. All of them are from the younger cohort, retired at a relatively young age and have a low to moderate pension income. They are all married and not overburdened by grandchild care chores. All these conditions allow them to take full advantage of the new freedom in retirement by being engaged in hobbies, something they did not get to do in earlier years. Also, the collectivist value and the drive for struggle they upheld while they were young seem to be reinforced in retirement, which made them so devoted.

Ms Peng (66) started taking dance classes just before retirement and now she is a professional dancer. Thanks to family support, she takes dance seriously and prioritises her regular dance rehearsal among all the possible activities in life. She reflects on her continued interest in dancing and perceives retirement as providing an opportunity to make up for the regret and achieve her dream in youth: ‘It was during those times that nobody wanted their kids to do arts. I felt it was a pity, and it's the biggest regret of my life’. Now dance has shaped her enthusiasm towards life: ‘You feel that living in this world is a happy thing, and every day is so beautiful.’ Hours devoted to leisure activities take up a great portion of time and the activities have become a major source of meaning in the retirement life for people like Ms Peng. They are highly motivated to carry on their current life routines, and their leisure pursuit has sometimes reshaped their outlook on life in a positive fashion.

*Versatile engagers* are those who basically love being with people and are extensively involved in various activities for the purpose of social interaction or health promotion. People from this category explore different possibilities, but they do not necessarily delve into them as the ‘devoted pursuers’. Lawton (1993) distinguishes between experiential, developmental and social meanings of leisure. It is the social leisure that is most relevant in the case of versatile engagers, and leisure experience creates a context for social interaction. Eight informants including six women and two men share the characteristics of this pathway. It seems that women are looking for more social interaction than men in retirement.

For Ms Ai (80), life is filled with choices in retirement, something that was greatly restricted for most of her life. Her hobbies and activities include: gardening, keeping golden fish, calligraphy, painting, taking senior university classes, photographing, walking, travelling, cooking, making fragrant sachets and playing mahjong. Her social network consists of family, relatives, previous students and colleagues, and she edifies her sense of self through family and social interactions. She intentionally seeks and reaches out to others. For people with such characteristics, pleasure in activity engagement is often expressed in terms of companionship sought or social support received.

*Productive non-stoppers* are those who are career-orientated – continually working towards maintaining a sense of self-achievement and social usefulness – and/or see busyness as a desirable goal in life. Retirement does not matter for these people since their work role is maintained through re-employment. It should be noted that continual work after retirement does not necessarily put a person into this category: it depends on the person's motivation for seeking and assuming the role of employee, whether it is to obtain a sense of achievement or a way of distraction to escape from the daily routine at home. Five informants portray themselves as work-orientated non-stoppers. They are better educated (including a rare college graduate) and have held fulfilling professional careers prior to retirement – office jobs that require specialised skills, which are still highly valued in reforming China. As commented by Mr Su (74), ‘the key is … you as an individual must have some knowledge so that others would come to your door even when you're sitting at home’.

Ms Tang (82) is still working in her early eighties, which is an extreme exception under the mandatory retirement system. Because of her expertise and achievement, she is among the very few State Council Special Allowance awardees nationwide. She is very expressive when explaining how the system of medical talent is operating, and she shows her professional identity throughout the talk. She has a fixed schedule every day and reading that facilitates work takes up most of her time. Mr Ma (74) retired from a design institute and was immediately re-employed by his previous work unit. A new work position appeared because of the state's need and it provides individuals with work opportunities after retirement. He emphasises his skills and experience in the field and shows a strong work identity: ‘I'm still working, so I feel I still have some value in this society … I just feel I'm still needed by society.’ It is evident that for productive non-stoppers, their devotion to productivity and desire to serve people and the society is also a reflection of the strong work ethics emphasised in the Maoist ideology in their earlier years.

*Lifelong learners* are those who continue to learn for the purpose of acquiring knowledge or keeping up with the times. These people proactively seek information and technology so as not to be left behind by the society. Interestingly, six informants including five women share the characteristics of such a pathway.

Ms Tang (82) is open to changes, and is not afraid to be taught by the younger generations. She sees young ones as bearers of knowledge. She describes her lifestyle as being a homebody using the Chinese term *zhai*, a popular expression among the young generation. Apparently, she is not segregated from popular culture and remains socially engaged. Being a determined lifelong learner, Ms Gao (57) decidedly resists the ageist attitudes of people around her: ‘Sometimes, when my friends called me to play mahjong with them, I said I was busy. They responded, “Look how old you are! Don't act like the young.”’ Ms Chang (50) points out the importance of keeping an open and receptive mind to new things, taking the initiative in catching up with the world and thus winning respect from the young: ‘You can't take getting old as an excuse for quitting learning … Once you keep learning and accepting new things, the young people will regard you as an elder who is respectable.’

On a related note, although senior university was originally designed to serve the privileged ageing population in China, including retired cadres or retirees from government organisations and public institutions, it has become more open and inclusive to the public in recent years as a consequence of economic reform (Chui 2012). According to Ms Li's (63) experience of attending senior university after retirement, a variety of classes are offered at different levels, and teachers are usually retired professionals in that field. The tuition is affordable even for the working class. Similarly, Ms Ai (80) attended senior university centred around her hobbies motivated by lifelong learning and socialising. She is a believer that ‘You're never too old to learn.’ An age-inclusive social network functions as an empowering social and psychological resource for her to enjoy a meaningful later life.

*Spiritual seekers* are those who stay aloof from materialistic pursuit and regard religious practice or spiritual exploration as essential to building meaning in their life. The four informants belonging to this category all grew up with Mao's ideology and atheism. Retirement and the new freedom to pursue religious beliefs in reforming China have brought them the possibilities for wisdom and spiritual growth. The overwhelming culture of materialist consumerism in urban China among the younger generations has also made it necessary for some elders to turn to religions for seeking a peaceful mind. Ms Luo (55) has a negative perception of social change and passes a moral judgement on the different values of different generations. She misses the ‘good old days’ under the planned system and criticises the over-abundance of goods and endless desires brought about by the economic reform. She connects her religious experience back to childhood memories. As religious freedom is regained in reforming China, it opens new opportunities for these retirees.

Religious beliefs and practices bring positive change in their lives and make them more prone to lead a life away from secular pursuits. In fact, people with no religious affiliation can also enjoy a rich intellectual world and live a transcendent life. Mr Bai (60) shares his life philosophy and describes his unruffled state of mind. He has an underlying idea of living in the present, to not waste time. He has the ability to see the duality of things and adopts a proactive approach to life: ‘Living in society, make changes if you can, or adapt to it if you can't change it … In every situation, you should be able to advance or retreat.’

*Generous helpers* are those who are warm-hearted in nature and love doing good for others by helping whenever an opportunity arises. These people find pleasure in offering their knowledge, skills or care to those in need without expecting anything in exchange. Six informants reflect such shared traits somehow, and most of them are from the working class and have a lower educational background.

Ms Dai (56), Mr Cao (64) and Ms Zheng (64), all retired workers, show their interest in volunteering within the neighbourhood, though structural opportunities are often lacking. They recognise the need for programmes to make the quality of life of older adults better and sustainable and hope for a more active role for the community in providing services and activities. It seems that the collectivist ideology in earlier life, such as following the crowd and waiting for orders from the CCP organisations, are still influencing their decision-making process in later life. These people count on structural opportunities, something they were used to during the Mao era, to make a change in their life. The role of community seems to be crucial in mobilising these individuals. Li (2010) also finds that most of the older Chinese volunteers in her sample selected in Jinan, China count on their Community Residents’ Committees for volunteer opportunities.

Mr Su's (74) lives on a high pension income. Giving back to the rural community he grew up with has central meaning in his retirement life. His hometown is in the countryside, quite close to where he is living, so he gets to visit it frequently, seeing his relatives and generously helping the local peasants to improve their standard of living: ‘When I was back in the countryside, the peasants were growing crops, so I taught them how to make more money … the peasants were building a house, so I helped them design the structure and calculate the cost.’ He loves to share his knowledge with the local peasants and offer help. For generous helpers, altruism and ‘serving the people’, an ideology learnt from the Mao era, seems still relevant in meaning-making in retirement.

Although history leaves its imprint on all of the informants and reminiscence constitutes a meaningful part of the ageing experience, the different pathways in retirement through the practice of claiming and exercising agency reflect the variability in how individuals actually age. In most of the cases, human beings are indeed active agents in constructing their present life worlds (Gecas 2004) by drawing on the resources available, though for some, perceived constraints may hinder the revival of dormant agency which is moulded by their earlier experiences.

## Discussion

The present study explores the retirement experiences of today's Chinese elders by focusing on their agency, connecting their current life situations to their early life experiences and placing them within the context of drastic historical changes.

### Historical embeddedness and changing values

The impact of historical contexts resonates in the current study of older Chinese inasmuch as they consistently narrate a collective memory of earlier lives that were beyond personal control and filled with hardships. Being raised in the Mao era and educated with the national ideology of planned socialism and collectivism, there had been very limited room for individualism – the core value of Western culture – and personal choices had been dwarfed upon confrontation with the dominating social structures in their earlier life. Through viewing the present self in relation to the choiceless past, reminiscence seems to help the informants carve out who they are now and create a sense of self-integrity in their later life. As a universal experience in later life, reminiscence may benefit the socio-psychological state in later life through mechanisms of ‘identity-forming and self-continuity; enhancing meaning in life and coherence; preserving a sense of mastery; and promoting acceptance and reconciliation’ (Bohlmeijer *et al*. 2007: 291). More importantly, these retirees tend to perceive the difficult past as a reference guide for relative appreciation of happiness and a contributor to their resilience in later life. George (1996) highlighted the lasting impact of historical events on one's subjective wellbeing, reflected in life satisfaction, personality, identity, morale, attitudes and behaviours in later years. Rather than living in the past, the informants in this study reached a coherence of meaning in life through bridging the past and the present, and raising an awareness of historical comparison, or relative appreciation of happiness or hardship. Both Liang's (2011) study of one particular birth cohort and the current study which includes different cohorts have demonstrated that such historical influence can and does carry into the process of meaning-making and identity formation in retirement or old age in China.

Ageing itself is a process of migration throughout time: older persons are living in a culture different from the one in which they grew up (Westerhof 2010). The context of reforming China has added an extra layer of change in the ageing process for these Chinese retirees. However, one important research area often neglected is the impact of social change on cultural meanings, as reflected in individuals’ perceptions and interpretations of social phenomena (George 1996). Yang (2003) argued that nostalgia among the Cultural Revolution generation not only constitutes an essential means of identity construction but also is a form of cultural resistance against the present values dominated by materialism, consumerism and instrumental rationality, as well as a critique of moral decline embedded in modernity. As the present study has shown, traditional culture, ideology, morality and values may lead to a negative perception of social change for some Chinese elders, who eventually retreated from society and become ‘passive time-riders’. However, most of these retirees took more proactive paths and demonstrated resilience in adapting to the social changes.

### The power of agency and its variations

Agency among the Chinese retirees emerged as an essential concept for this study. Although there has been a growing interest in the role of agency among older people in the West, ageing studies in China have predominantly focused on dependency and passivity. Psychologically, the core of agency is self-efficacy (Bandura 1997), the sense of having control over one's life circumstances. Agency is embodied in cognitive and affective aspects ‘such as regulating perception, processing experience, memory, emotion, and motivation’ (Gecas 2004: 369). Sociologically, agency can be defined as personal choice, subjectivity, reflexivity and empowerment, as opposed to structure (Katz and Laliberte-Rudman 2005). In general, individuals with high self-efficacy perceive themselves as competent, effective and able, while those with low self-efficacy see themselves as powerless, helpless and fatalistic (Bandura 1997). The belief in self-efficacy is the determinant of passive reaction or proactive action. From a lifecourse perspective, agency in later life cannot be studied alone without reference to individuals’ life circumstances within particular social, political, historical and cultural contexts.

The variation caused by the different locations of cohorts among the informants accounted for some, but not all, of the differences in their exertion of agency. Even the people from the same cohort are not homogenous; their different social locations, such as gender, social class and religious affiliation, have affected their perceptions and interpretations of retirement and old age. In general, people with high levels of physical, material, social and psychological resources are more likely to have a positive experience of ageing (Steverink *et al.* 2001) and thus displaying high levels of agency. In the present study, resources that stimulate proactivity and facilitate retirement transition include hobby-based leisure activity, professional knowledge and skills, family support, social network, religious belief and practice, environmental amenities (*e.g.* physically, easy access to open space; socially, age-integrative environments) and positive outlook on life (*e.g.* openness to differences, associating ageing with human growth). By contrast, constraints that generate passivity and hinder the pursuit of meaningful goals in later life include health decline, financial strain, adherence to traditional ideology and education, environmental disadvantage (*e.g.* lack of open space, age-segregated environment, ageist culture) and passive outlook on life (*e.g.* negative perception of ageing and social change).

Lifecourse is not a linear or unidirectional process of advantage or disadvantage accumulation. By way of example, Elder (1994, 1998) emphasised earlier hardships do not necessarily transfer to disadvantaged socio-economic status or adverse health outcomes by noting that some children who suffered poverty during the Great Depression ended up as high achievers and in good health in adulthood. Similarly, Zhou and Hou (1999) suggested that ‘hardship’ in youth experienced by the ‘sent-down’ generation in China might also have a positive impact on their determination to thrive in society in middle age. As illustrated by these Chinese informants, the initial ‘vulnerability’ or ‘disadvantage’ resulting from limited personal choices in early life did not necessarily lead to diminished freedom in planning post-retirement life, nor to lessened capacity to exercise human agency. In other words, their earlier life experiences of centrally planned economy and collectivist ideology did not necessarily put them in a disadvantaged position in retirement, making them feel powerless, aimless or even victimised in an era of ravaging market economy in which structures are diminishing while personal choices are expanding. Rather, they could make choices and adaptations facing the social changes and the challenges of ageing, and discover and exercise agency through actively bridging the past and the present and constructing meaningful pathways in later life. Although some, the ‘passive time-riders’, are still beset with the perception of retirement as a ‘roleless role’ (Rosow 1974) – their thinking and behaviours must be understood in relation to their earlier experiences of growing up in the socialist and collectivist climate where individual options were rare – the rest attempt to reconstruct retirement towards a life-stage with potential roles that have intrinsic values.

### Practical implications

Findings from this study have implications for developing policies, designing programmes and providing services that take advantage of the agency of Chinese elders to improve their wellbeing. First, reminiscence can be implemented as a therapeutic intervention programme (Merriam 1993) for older Chinese, or developed as a means to enhance mutual intergenerational understanding and relationships (Mercken 2003). On a personal note, for the younger Chinese generations, listening to older generations’ stories about a shared past can be a precious learning experience to gain new insights into history and redefine oneself.

Second, the present study has posed a good challenge to the extant literature that often ignores the agency among Chinese elders and opened new opportunities for policy makers and service providers to serve accurately the actual interest of the ageing population. The varying individually constructed pathways have identified potential new social roles in retirement and shed light on the opportunities that could be created at the level of social institutions. A wide range of roles for retirees that can be promoted at the service level includes but is not limited to the engagement in leisure activity, social activity, gainful activity, lifelong learning, religious or spiritual activity, and volunteering. Establishing and improving services and programmes (*e.g.* skill-sharing, retirement-planning, volunteering) at community-affiliated senior activity centres and offering classes that cater to older learners on university campuses are just a few of the many possible ways to enhance retirees’ quality of life.

Third, at the policy level, strengthening resources and alleviating constraints that condition the exercise of human agency, and mobilising the passive time-riders and facilitating the rest of the active time-users is pivotal in improving the wellbeing for Chinese retirees. To be noted, ‘outlook on life’ and ‘environment’ are the two domains that migrate between ‘resource’ and ‘constraint’ easily. Ageing is a meaning-making process within a particular cultural context. Promoting a positive social psychology that fights against ageism – strengthening age-integrative, barrier-free designs of public infrastructures to accommodate needs across all ages, and cultivating awareness that ageing is a lifelong process concerning everyone – can stimulate cultural change.

### Conclusion

Despite limitations with the present study, such as lack of geographic diversity and one-time interviews, this qualitative study is able to discover the shared experience of encountering retirement as a new life-stage through reminiscing and exerting agency, and at the same time to reveal the heterogeneity in constructing a meaningful pathway of later life among Chinese retirees. The findings from this study have refuted the public discourse that often portrays Chinese elders as passive care recipients or helpless dependants. As emphasised by Gilleard and Higgs (2000), positioning old age or elders as a deserving subject equivalent to poverty and exclusion in the domain of social policy is now changing, and research topics on meaning and identity are emerging to meet the social need. What is central to the issue is how individuals express and interpret their own ageing. We hope research on Chinese elders can soon catch up with such recognition: Chinese elders are not a homogenous group who are vulnerable, dependent and helpless, and often marginalised or even alienated from the overall population; instead, they are human beings who are active agents, capable of making choices and adaptations, within particular socio-historical structures.

## NOTES

1 In Xi'an, the number of older people aged 60 and over had reached 1.2 million by 2009, accounting for 14.9 per cent of the total population (Ma 2011).

2 Exceptionally, because of the legislated retirement age and the approximate age at the birth of first grandchild, age 50, rather than 60 or 65, is often regarded as the onset of old age in China (Davis-Friedmann 1991). The retirement age is 60 years for male cadres and workers, 55 years for female cadres and 50 years for female workers; for those who are working in arduous conditions, men can retire at age 55 and women can retire at age 45. A minimum of a ten-year accumulated work history is required by the legal retirement system in China (China's Ministry of Human Resources and Social Security 1999).

3 The so-called ‘internal retirement’ policy (Giles *et al*. 2012) was introduced for the purpose of lowering the cost of human capital, asking older workers to withdraw from their positions ahead of the mandatory retirement age, usually within the range of five years. Some study participants had been initially internally retired with a limited monthly stipend (usually lower than the pension income) and then transferred to full retirement with pension benefits. Workers who had been simply laid-off were not targeted in the current study with the consideration that they are more similar to the unemployed, though internal retirement in some cases could be regarded as being laid-off.

4 In 2011, when the interviews were conducted, US $100 was equal to approximately 646 Chinese yuan.

5 The educated urban youth were sent to mountainous or farming areas to learn from the workers and farmers.

6 Deng Xiaoping is considered to be the architect of China's economic reform.

7 Between 1958 and 1961, in order to speed up China's industrialisation, the Great Leap Forward attempted to make incredible strides in production, but the goals turned out to be unrealistic and the human cost was enormous.
